# *Aspergillus fumigatus* Influences Gasdermin-D-Dependent Pyroptosis of the Lung via Regulating Toll-Like Receptor 2-Mediated Regulatory T Cell Differentiation

**DOI:** 10.1155/2021/5538612

**Published:** 2021-06-14

**Authors:** Wei Yan, Yi-si Zhao, Ke Xie, Yu Xing, Fang Xu

**Affiliations:** ^1^Department of Critical Care Medicine, The First Affiliated Hospital of Chongqing Medical University, Chongqing, China; ^2^Forensic Medicine and Biomedical Informatics Research Room, Chongqing Medical University, Chongqing, China

## Abstract

**Purpose:**

*Aspergillus fumigatus*, as an opportunistic fungus, has developed a series of escape mechanisms under the host's immune response to obtain nutrients and promote fungal growth in the hostile environment. The immune escape of pathogens may be through suppressing the inflammatory response mediated by regulatory T cells (Tregs). The aim of this study was to explore whether *A. fumigatus* influences Gasdermin-D-dependent pyroptosis of the lung by regulating Toll-like receptor 2-mediated regulatory T cell differentiation.

**Methods:**

Collect peripheral blood from patients with *A. fumigatus*. ELISA kits we used to detect the expression levels of IL-1*β*, IL-6, IL-2R, and IL-10 in the serum and flow cytometry to detect the percentage of CD4^+^CD25^+^Foxp3^+^ Tregs in the patients' peripheral blood mononuclear cells (PBMCs). The mouse model of *A. fumigatus* infection was constructed by tracheal instillation. The pathological changes in the lungs of the mice were observed under a microscope. The fungal load in the lung tissue was determined by the plate colony count. ELISA kit was used to detect the lung tissue homogenate proinflammatory cytokines TNF-*α*, IL-6, CCL2, and VEGF. Q-PCR was used for the detection of the expression of Foxp3 and TLR2 genes in the lung. Western blot was used for the detection of the expression of TLR2, Gasdermin-D (GSDMD), IL-1*α*, and IL-1*β* in the lung. Flow cytometry was used to detect splenic CD4^+^CD25^+^FOXP3^+^ Tregs. Using magnetic beads to extract CD4^+^ T cells from mice spleen, the effects of *A. fumigatus* conidia or TLR2 inhibitor (C29) to differentiate CD4^+^ T cells in vitro were tested.

**Results:**

The expression of Foxp3 and TLR2 in the lung tissue of mice infected with *A. fumigatus* increased, and we observed that the proportion of Tregs in both *A. fumigatus* infection patients and mice was upregulated. After using the CD25 neutralizing antibody, the number of Tregs in the mice spleen was significantly reduced. However, lung damage was reduced and the ability to clear lung fungi was enhanced. We found that the Tregs in TLR2^−/−^ mice were significantly reduced and the nonlethal dose of *A. fumigatus* conidia did not cause severe lung damage in TLR2^−/−^ mice. Compared with that of wild-type mice, the fungal burden in the lung of TLR2-deficient mice was reduced and the knockout of TLR2 changed the expression of GSDMD, IL-1*α*, and IL-1*β* in *A. fumigatus*. In in vitro experiments, we found that the inhibition of TLR2 can reduce Treg differentiation.

**Conclusions:**

*A. fumigatus* triggers CD4^+^CD25^+^FOXP3^+^ Treg proliferation and differentiation by activating the TLR2 pathway, which may be a potential mechanism for evading host defenses in *A. fumigatus*. This effect can modulate GSDMD-dependent pyroptosis and may partly involve TRL2 signaling.

## 1. Introduction

*Aspergillus fumigatus*, as an opportunistic fungus, is one of the common conidia species in the environment [1, 2] that can cause lung and systemic infection in humans [3, 4], with approximately 200000 cases of invasive Aspergillus (IA) patients each year globally [5, 6]. Regulatory T cells (Tregs) have been shown to control the host's inflammatory response. Studies have found that mice have a significant increase in Tregs after infection with *A. fumigatus* [7] and showed the conventional T lymphocyte response in the amount and target specificity [8–10]. Treg cells can inhibit excessive tissue inflammation by inhibiting Th1 and Th17 responses during the first few days after infection by *A. fumigatus* [9]. However, Tregs also promote immune tolerance and immune escape by restraining the body's immune response increasing the sensitivity of bacterial infections [11]. TLR2 is a member of the pattern recognition receptor (PRR) family and triggers host responses [12, 13], activated by either *A. fumigatus* stimulation. Judging by the current analysis and research, in addition to innate immune cells like killer cells, dendritic cells, and macrophages exhibiting TLR2, the same TLR2 expression is seen on several adaptive immune cells like CD4^+^, CD8^+^ T cells [14], and CD4^+^Foxp3^+^ Tregs [15]. The proliferation of CD4 ^+^ Foxp3^+^ Treg cells can be induced by the TLR2/MyD88 pathway in dengue infection [11]. In a mouse test to analyze infection caused by *Candida albicans*, reducing the expression of TLR2 lowers the quantity of CD4^+^CD25^+^ Treg cells and decreases the fungal burden [16]. Macrophages had a lowered secretion of proinflammatory cytokines in response to *Aspergillus*-stimulated production of IL-10 via TLR2-dependent mechanisms [17].

Thus, we hypothesized that *A. fumigatus* stimulates TLR2 signal activation to induce an increase of CD4^+^CD25^+^FOXP3^+^ Tregs, thereby mediating inflammatory environment changes in the lung and promoting fungi' persistence. To clarify the assumption, we analyze seven clinical samples of *A. fumigatus* infection. Then, we created a mouse model of pulmonary infection of *A. fumigatus* in wild-type mice and TLR2^−/−^ mice and through in vitro experiments to detect the role of TLR2 in the differentiation of CD4^+^ T cells.

## 2. Materials and Methods

### 2.1. Clinical Sample Collection

Fourteen blood samples were collected in the study. Seven samples of the infection group were from adult patients with *Aspergillus fumigatus* infection in the First Affiliated Hospital of Chongqing Medical University, and seven healthy samples were included as the control group of the study. Every participant provided their consent before sample collections, and the Clinical Research Ethics Committee of the University approved the protocol. The number is Lot 2020-850.

### 2.2. Human Peripheral Blood Mononuclear Cell (PBMC) Isolation

EDTA tubes were utilized to store the collected blood samples. The samples were centrifuged at 2500 rpm for 8 min by density gradient (Histopaque, Sigma). According to the instructions, we used the Human Peripheral Blood Lymphocyte Separation Solution Kit (TBDsciences) to obtain lymphocytes.

### 2.3. Human Serum Cytokine and Peripheral Blood Treg/CD4^+^ Measurements

The blood samples were centrifuged at 2000 rpm for 15 min at 4°C to obtain supernatant for serum. The levels of IL-1*β*, IL-6, IL-2R, and IL-10 in the serum and Treg/CD4^+^ (the percentage of CD4^+^CD25^+^Foxp3^+^ Tregs in CD4^+^ T cells) in the PBMCs were analyzed at the Clinical Molecular Testing Center of the First Affiliated Hospital of Chongqing Medical University for measurements.

### 2.4. Animals

C57/BL6 mice (male, 6–8 weeks, 17–24 g) used in our experiment were acquired from the Laboratory Animal Center of Chongqing Medical University. TLR2 knockout mice with a C57/BL6 background were purchased from Jackson Laboratory. All procedures were approved by the Institutional Animal Care and Use Committee at Chongqing Medical University.

### 2.5. Strains of Fungi and Conditions for Cultivation

The strain of *A. fumigatus* used was Af293 with the required specifications for infections and cultivation as previously described [18]. Briefly, conidia were matured on Sabouraud Dextrose Agar plates for seven days at 37°C and 5% CO_2_. To prepare a spore suspension, rinse with 10 mL sterile PBS containing 0.1% Tween 20 and gently scrape the *Aspergillus* colonies on the Petri dish [19]. Then, filter through eight layers of sterile gauze. After adjusting the fungal suspension to the desired concentration with a hemocytometer, the conidia suspension was stored at 4°C.

### 2.6. The Mouse Model with *Aspergillus fumigatus* Infection and Tissue Sample Collection

Mice were mildly anesthetized and then placed in a flat position and administered intratracheally at a concentration of 50 *μ*l of 1 × 10^7^ viable spores while maintaining an upright position to be used as the study model for infections caused by *A. fumigatus* [20]. Within 1 to 2 hours after injection, the mice recovered completely and had a healthy appearance. The mice were kept at the SPF laboratory and euthanized at 24 and 72 h after the operation. Blood was collected retro-orbitally. Lung tissue and spleen of mice were obtained for subsequent research. Spleen was taken out of mice and gently ground with a mesh screen to obtain spleen cells for subsequent research.

### 2.7. Blockade of Treg Cells In Vivo

Mouse CD25/IL-2R alpha antibody (AF2438, R&D Systems, Minnesota, MN, USA) was used to suppress Treg cells. One hour before *A. fumigatus* infection, each mice in the inhibitor group was intraperitoneally injected with 20 *μ*g of mouse CD25/IL-2R alpha antibody, and control mice were injected with antibody rat IgG1 [21].

### 2.8. Histopathology

Samples of the lung were fixed in 4% formaldehyde. Sectioning was done after the samples were embedded in paraffin wax. Grocott's methenamine silver (GMS) was utilized to stain the lung samples for the detection of fungus. For the histological analysis procedure, lung samples were stained with either hematoxylin and eosin (H&E). Analyzed through the use of COOLSCOPE digital light microscope (Nikon Co., Tokyo, Japan), lung injury was scored according to criteria defined by Mikawa et al. [22] as follows: (1) alveolar hyperemia, (2) hemorrhage, (3) interstitial or aggregation of interstitial or neutrophils, and (4) thickening of the alveolar septum or hyaline membrane formation. Pneumoniae pulmonary infection scores were approximated through the method by the scoring standard published by Cimolai et al. [23]. The scoring standard is based on (1) the infiltration degree of inflammatory cells around the trachea and bronchiole 0–3, (2) quality of trachea and bronchiole infiltrate 0–3, (3) infiltration degree of inflammation in trachea and bronchiole cavity 0–2, (4) infiltration of inflammatory cells around blood vessels, degree 0–3, and (5) inflammation of the lung parenchyma which involves the range 0, 3, and 5. The severity of the inflammation is directly proportional to the magnitude of the score.

### 2.9. In Vivo Quantification of Viable Conidia

The fungal burden in the lungs of mice was determined by the plate colony counting method. Separate mouse lungs aseptically, weigh their wet weight, add ice PBS, homogenize the tissue, and dilute the tissue proportionally. Each concentration gradient (10^−1^ and 10^−2^) was added to the sandcastle plate medium, and each concentration gradient was inoculated with two dishes and cultivated at 37°C for 72 hours. Count the colonies and multiply by the dilution factor.

### 2.10. Cytokine Measurements

Lung tissue homogenates (10-fold dilution) of WT mice and TLR2^−/−^ mice were collected. According to the instructions, tumor necrosis factor (TNF)-*α*, IL-6, VEGF, and CCL2 were measured following the enzyme-linked immunosorbent assay kit (ELISA) as measured (4A Biotech, China).

### 2.11. Extraction of RNA, Synthesis of cDNA, and Real-Time Quantitative PCR

TRIzol (TAKARA BIO, Tokyo, Japan) was used to extract total RNA from lung tissue and measure the RNA concentration. The experiment pays attention to prevent contamination of exogenous RNase. The specific experimental procedures follow the instructions of TB Green® Premix Ex Taq™ II (Tli RNase H Plus) (TAKARA BIO, Tokyo, Japan). The added specific primers to the reaction system to perform RT-PCR include TLR2, Foxp3, and glyceraldehyde triphosphate dehydrogenase (GAPDH). The primer sequences were as follows: TLR2 forward 5′-GATGAAGTCAGCTCACCGAT-3′; reverse 5′-ACAGTTCCAAGATGTAACGC-3′; Foxp3 forward 5′-CCTATGCCACCCTTATCCGATG-3′; reverse 5′-CGAACATGCGAGTAAACCAA-3′; GAPDH forward 5′-GGACACTGAGCAAGAGAGGC-3′; and reverse 5′-TTATGGGGGTCTGGGATGGAA-3′. Using a 25 *μ*l system, add TB Green Premix Ex Taq II (Tli RNase H Plus) (2x), forward primer, reverse primer, DNA template, and RNase-free dH_2_O according to the instructions. Adopting a two-step PCR reaction program, the 2^ΔΔ*C*(*t*)^ approach is used to determine the expression of the relative target gene.

### 2.12. Western Blotting Assay

Utilizing a homogenizer, homogenization of the lung tissue in 1 ml 50 mM Tris-HCl (pH 7.8) containing 15% glycerol, 150 mM NaCl, 0.1% Tween-20, and protease inhibitors was done and followed by centrifugation. The protein concentrations were estimated through the use of a bicinchoninic acid (BCA) Protein Assay Kit (Beyotime, Shanghai, China). The supernatant (total protein) was separated with sodium dodecyl sulfate-polyacrylamide gel electrophoresis (SDS-PAGE) and blotted onto a polyvinylidene difluoride membrane. The membrane was blocked with 5% (*w*/*v*) skimmed milk and then incubated with TLR2 antibody (diluted at 1 : 300), Foxp3 (diluted at 1 : 1000) antibody, Gasdermin-D antibody (diluted at 1 : 1000), IL-1*α* antibody (diluted at 1 : 1000), IL-1*β* antibody (diluted at 1 : 1000), or GADPH antibody (1 : 2000) at 4°C for 14–17 h, and then, the horseradish peroxidase-conjugated secondary antibody (diluted at 1 : 5000~8000) was reacted at 37°C for 1 hour. The membrane is exposed to enhanced chemiluminescence (ECL) reagents. Use ImageQuant TL software to detect protein expression.

### 2.13. CD4^+^T Lymphocyte Isolation, Proliferation, and Differentiation into Treg Cells In Vitro

The conidia of *A. fumigatus* were heat inactivated after heating at 65°C for 60 minutes. Sabouraud Agar is applied to test the viability of these conidia. The study indicates that the reagents used should have 1 × 10^7^/ml [24]. Spleen samples should be collected from C57/BL6 mice according to the previous method [25]. CD4^+^ T lymphocytes were isolated by the EasySep™ Mouse Naive CD4^+^ T Cell Isolation Kit (STEMCELL). About 5 × 10^5^ CD4^+^ T cells are inoculated in each well of the 48-well plate. The medium RPMI 1640 added 50 ng/ml transforming growth factor-*β* (TGF-*β*) (PeproTech), 5 *μ*g/ml anti-mouse CD3 (eBioscience), 2 *μ*g/ml anti-mouse CD28 (eBioscience),10 ng/ml cytokines IL-2 (PeproTech, Rocky Hill, NJ, USA), 50 mM *β*-mercaptoethanol (Macklin, Shanghai, China), and 2 mML-glutamine (STEMCELL Technologies, Vancouver, Canada), with or without C29, incubated at 37°C, 5% CO_2_ for 3 days, and then performed flow cytometry detection, with each group repeating 5 times. C_16_H_15_NO_4_ (C29) (MCE, New Jersey, USA) was dissolved in DMSO as 50 mM stock solution [26].

### 2.14. Flow Cytometry

The cultured T cells, isolated PBMCs, and splenocytes to be tested were incubated in the dark with fluorescent antibodies to determine the percentage of CD4 ^+^ CD25^+^ Foxp3 ^+^ Tregs in CD4^+^ T cells. According to the manufacturer's instructions, the collected cells are washed with PBS, centrifuged to pellet, and then stained with antibodies (anti-CD25-phycoerythrin-PE, anti-CD4-FITC, and anti-Foxp3-APC) and a Fixation/Permeabilization Kit (eBioscience)) was used in the dark for flow cytometry detection. At least 10^5^ cells were collected and detected with a FACS flow cytometer (Becton, Dickinson), and data analysis was done by FlowJo software V10.

### 2.15. Statistical Analyses

Statistical analysis was done using SPSS 20.0 (IBM, Armonk, NY, USA) and GraphPad Prism 8.0 (GraphPad Software, San Diego, CA, USA). All experimental data were expressed as either mean ± standard deviation. Experimental data were assessed with Student's unpaired two-tailed *t* test, one-way ANOVA, or two-way analysis of variance (ANOVA) attended by the Tukey Post Hoc test. *p* < 0.05 was regarded as statistically significant.

## 3. Results

### 3.1. *Aspergillus fumigatus* Infection Causes Lung Damage in Immunocompetent Mice

**Figure 1:**
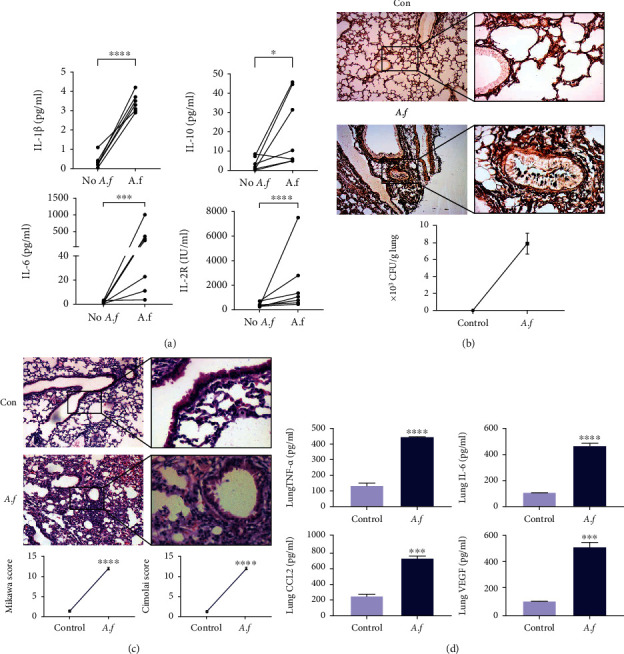
*Aspergillus fumigatus* infection caused the pulmonary inflammatory response. (a) IL-1*β*, IL-6, IL-10, and IL-2R are elevated in the serum of patients infected with *A. fumigatus* (*n* = 7). (b) C57BL/6 mice were administered intratracheally at a concentration of 50 *μ*l of 1 × 10^7^ viable spores and monitored for 3 days (*n* = 5/group). Grocott's methenamine silver (GMS) and lung colony-forming units (CFUs) (*n* = 5/group). (c) H&E staining of the lung tissues of *A. fumigatus*-infected mice at 100x and 400x magnification, compared with the noninfected control, alveolar hemorrhage, and inflammatory cell infiltration were more after *A. fumigatus* challenge. (d) TNF-*α*, IL-6, CCL2, and VEGF expression levels in lung tissues of mice were detected by ELISA. *A. fumigatus* upregulated the production of proinflammatory cytokines/chemokines in lung tissues, including TNF-*α*, IL-6, CCL2, and VEGF (*n* = 3/group). Experiments were done at least three times. ns: not significant; ^∗^*p* < 0.05, ^∗∗^*p* < 0.01, ^∗∗∗^*p* < 0.001, and ^∗∗∗∗^*p* < 0.0001 by Student's unpaired two-tailed *t* test. Error bars represent SEM.

We found that IL-1*β* (*p* < 0.0001), IL-6 (*p* < 0.001), and IL-2R (*p* < 0.0001) raised among patient specimens infected with *A. fumigatus*; IL-10 was slightly increased after infection (*p* < 0.05, Figure 1(a)). Then, we established mice lung infection with *A. fumigatus*. The lung infection of *A. fumigatus* in mice was confirmed by GMS staining and the number of fungal colonies (Figure 1(b)). As shown in Figure 1(c), we observed that H&E of lung tissue sections showed infiltration of inflammatory cells in the bronchiole, perivascular, and vascular lumen. The degree of lung damage semiquantitative injury index includes hemorrhage, alveolar hyperemia, interstitial or neutrophil infiltration or aggregation, and severe inflammatory cell infiltration in the *A. fumigatus* pneumonia compared to the control mice. Appreciably enhancive quantities of inflammatory cells and increased lung histopathology Mikawa scores and Cimolai score were observed in the *A. fumigatus* infection group (*p* < 0.0001). Simultaneously, *A. fumigatus* significantly increases the concentration of chemokines or cytokines, including TNF-*α* (*p* < 0.0001), CCL2 (*p* < 0.001), IL-6 (*p* < 0.0001), and VEGF (*p* < 0.001) in the lung tissue of mice (Figure 1(d)).

### 3.2. Increase of Treg Cell Ratios after *A. fumigatus* Stimulated in the Lung of Immunocompetent Mice

**Figure 2:**
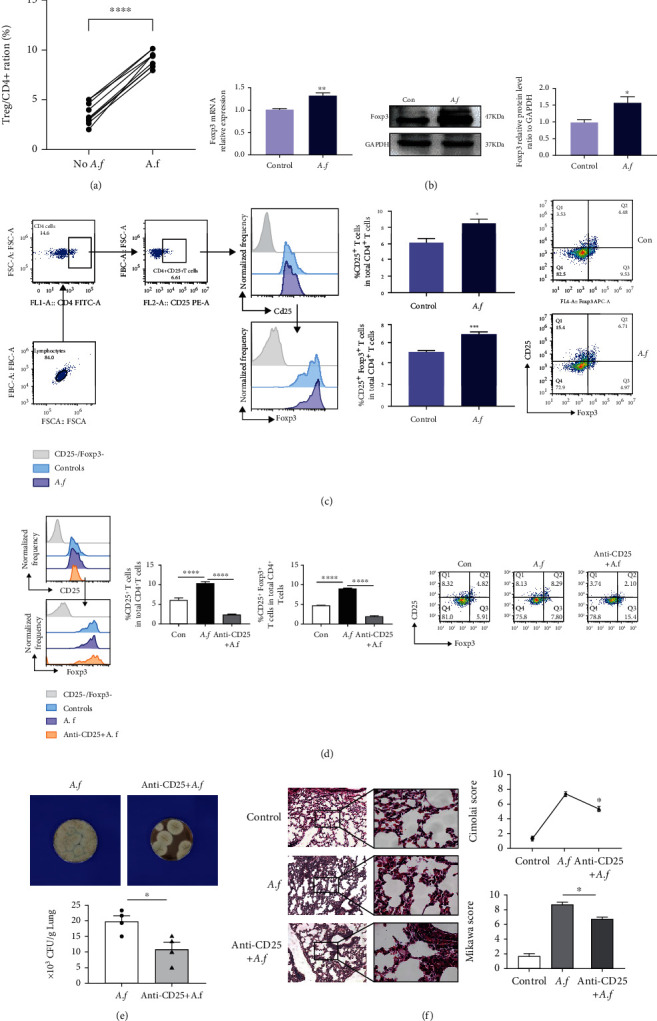
The persistent presence of fungi in lung injury induced by *Aspergillus fumigatus* is related to Tregs. (a) Increased CD4^+^CD25^+^Foxp3^+^ Treg proportion significant in PBMCs of patients infected with *A. fumigatus* (*n* = 7). (b) Foxp3 mRNA levels in the lungs were measured with q-PCR. Relative expression levels of the genes were expressed with the GAPDH housekeeping gene as an internal reference (*n* = 5/group). The expression of Foxp3 protein levels in the lungs was measured with Western blotting. Relative expression levels were expressed with the GAPDH as an internal reference (*n* = 3/group). (c) CD4^+^CD25^+^ T cells and CD4^+^CD25^+^Foxp3^+^ Tregs in the spleen were detected by flowcytometry, and FlowJo10 analyzed the proportions as prior described in Section 2. Increased CD4^+^CD25^+^ T cell and CD4^+^CD25^+^Foxp3^+^ Treg proportion significantly in the spleen of *A. fumigatus* infection mice (*n* = 5/group).Decreased susceptibility of mice to *A. fumigatus* infection after Treg depletion. (d) After pretreatment with CD25-neutralizing antibodies, the number of Treg cells in the spleen of mice with *A. fumigatus* infection group was significantly reduced, each group (*n* = 5/group). (e) Fungal load (colony-forming unit) after 1 day of infection (*n* = 4/group). Experiments were done at least three times. Data are presented as mean ± standard deviation. ^∗^Statistically significant difference (*p* < 0.05) against noninfected control. (f) Lungs from each experimental group were processed for histological examination after H&E staining. Lung injury scores were evaluated by the method described previously. ^∗^*p* < 0.05, ^∗∗^*p* < 0.01, ^∗∗∗^*p* < 0.001, and ^∗∗∗∗^*p* < 0.0001 by Student's unpaired two-tailed *t* test and the one-way ANOVA followed by the Tukey post hoc test. Error bars represent SEM.

As illustrated in Figure 2(a), the number of Treg cells in patients infected with *A. fumigatus* slightly increased (*p* < 0.0001). To further understand Tregs' involvement in pulmonary *A. fumigatus*, we studied the protein and mRNA levels of Foxp3 in the lungs post *A. fumigatus* challenge by real-time quantitative PCR and Western blotting. There was upregulated expression of Foxp3 in the lungs of mice who suffered *A. fumigatus* infection compared with controls (*p* < 0.05, Figure 2(b)). Simultaneously, we found that compared with noninfected mice, the CD25^+^ Tregs (*p* < 0.05) and CD25^+^Foxp3^+^ Tregs (*p* < 0.001) in CD4^+^ T cells of the spleen of mice treated with *A. fumigatus* increased significantly within 72 h after infection (Figure 2(c)). The above data suggested that the increased ratio of Treg cells was associated pulmonary *Aspergillosis*.

### 3.3. The Persistent Presence of Fungi in Lung Injury Induced by *Aspergillus fumigatus* Is Related to Tregs

To further clarify the role of Tregs in *A. fumigatus* infection, intraperitoneal injection of CD25-neutralizing antibody 20 *μ*g was used to inhibit Treg cells in WT mice. The quantities of CD25^+^ Tregs (*p* < 0.0001) and CD25^+^Foxp3^+^ Tregs (*p* < 0.0001) were decreased in CD4^+^ T cells of the spleen after being treated with CD25 antibody compared with those of the *A. fumigatus* infection group (Figure 2(d)). After *A. fumigatus* infection, we found that mice treated with CD25 antibody were observed to have a slightly lower load of *A. fumigatus* in their lungs compared with the *A. fumigatus* infection group (*p* < 0.05, (Figure 2(e)). As shown in Figure 2(f), a higher number of inflammatory cells and hemorrhage in the alveolus were discovered in mice treated with *A. fumigatus*. However, overall, after inhibiting Treg cells, the inflammatory cells around the blood vessels are slightly lower and there were mildly reduced lung histopathology Mikawa scores and Cimolai scores compared to those of IgG1-treated mice with *A. fumigatus* infection in the lung (*p* < 0.05).

### 3.4. TLR2 Is Increased in the Lung of Mice Treated with *Aspergillus fumigatus*

**Figure 3:**
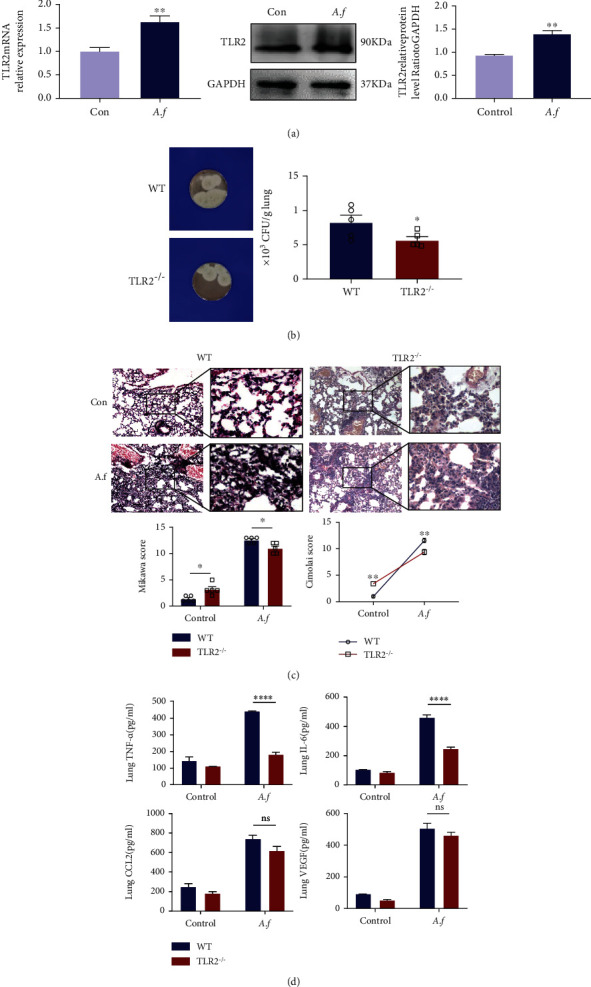
C57BL/6 mice and TLR2^−/−^ mice were infected with *A. fumigatus* spores and monitored for 3 days. (a) The expression of TLR2 protein in the lungs of mice which was infected with *A. fumigatus* for 3 days was detected by Western blot and analyzed by imager systems described in Section 2, and the mRNA levels of TLR2 were determined by q-PCR (*n* = 3/group). (b) Fungal load (colony-forming unit) after 3 days of infection (*n* = 5/group). (c) H&E staining of the lung tissues of *A. fumigatus*-infected WT mice and TLR2^−/−^ mice at 100x and 400x magnification, mainly infiltration of macrophages and monocytes in the lungs of TLR2^−/−^ mice with *A. fumigatus*, with alveolar congestion and hemorrhage. TLR2^−/−^ mice suffered from interstitial congestion and hemorrhage less obviously after *A. fumigatus* infection than WT mice. (*n* = 5/group). (d) TNF-*α*, IL-6, CCL2, and VEGF expression levels in the lung of mice were detected by ELISA. TLR2-deficient downregulated the production of proinflammatory cytokines/chemokines in lung tissues treated by *A. fumigatus*, including TNF-*α*, IL-6, CCL2, and VEGF (*n* = 3/group). Experiments were done at least three times. ns: not significant; ^∗^*p* < 0.05, ^∗∗^*p* < 0.01, ^∗∗∗^*p* < 0.001, and ^∗∗∗∗^*p* < 0.0001 by Student's unpaired two-tailed *t* test and the two-way ANOVA followed by the Tukey post hoc test. Error bars represent SEM.

TLR2 is one of the cell membrane receptors involved in *A. fumigatus* [27]. We measured the protein and mRNA levels of TLR2 in control and infected wild-type mouse lungs by Western blot and RT-PCR. The relative TLR2 mRNA expression levels were upregulated in the infected mouse lung compared to the control group (*p* < 0.01, Figure 3(a)). The TLR2 protein levels were also increased in the infected mouse lung compared to the control group (*p* < 0.01, Figure 3(a)). These results confirmed that the TLR2 expression is higher after *A. fumigatus* infection in the lung of mice.

### 3.5. TLR2^−/−^ Immunocompetent Mice Are less Susceptible to *Aspergillus fumigatus* Infection

To understand whether TLR2 is involved in Treg-mediated persistence in lung *A. fumigatus*, we infected TLR2^−/−^ mice with 1 × 10^7^ conidia which were inoculated and compared with wild-type mice; no deaths occurred in either of the two groups within 3 days (data not shown). Surprisingly, TLR2^−/−^ mice were observed to have a slightly lower load of *A. fumigatus* in their lungs compared with controls (*p* < 0.05, Figure 3(b)). As shown in Figure 3(c), histology of the lung showed that there is mainly infiltration of macrophages and monocytes in the lungs of TLR2^−/−^ mice with *A. fumigatus*, alveolar congestion, and hemorrhage. However, compared with TLR2^−/−^ mice, WT mice suffered from interstitial congestion and hemorrhage, more obvious after *A. fumigatus* infection, with neutrophil infiltration being severe. Assessing changes in lung tissue morphology, increased lung histopathology Mikawa scores (*p* < 0.05) and Cimolai scores (*p* < 0.01) were observed in the WT mice undergoing *A. fumigatus*. As illustrated in Figure 3(d), TNF-*α* and IL-6 were decreased in the lungs from TLR2^−/−^ mice compared with control wild-type mice with *A. fumigatus* infection (*p* < 0.0001). However, the expression levels of CCL2 and VEGF are not statistically different compared with WT mice.

### 3.6. TLR2 Plays a Crucial Role in Inducing the Proliferation of CD4^+^CD25^+^Foxp3^+^ Tregs in Lung Injury Induced by *Aspergillus fumigatus*

**Figure 4:**
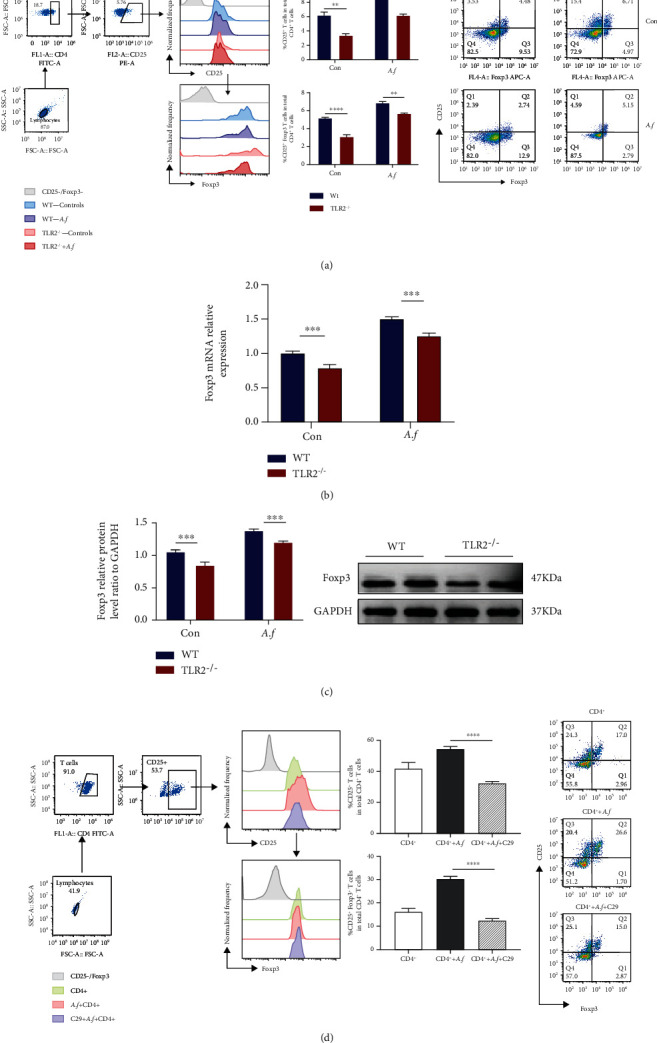
TLR2 affects the proliferation of CD4^+^CD25^+^Foxp3^+^ Treg in lung injury caused by *Aspergillus fumigatus*. (a) CD4^+^CD25^+^ T cells and CD4^+^CD25^+^Foxp3^+^ Tregs in the spleen were detected by flowcytometry, and the proportions were analyzed by FlowJo10 as prior described in Section 2. Reduced CD4^+^CD25^+^ T cell and CD4^+^CD25^+^Foxp3^+^ Treg proportion significantly in the spleen of *A. fumigatus* infection TLR2^−/−^ mice (*n* = 5/group). (b) Foxp3 mRNA levels in the lungs were measured with qRT-PCR (*n* = 3/group). (c) The expression of Foxp3 protein in lungs of C57BL/6 mice and TLR2^−/−^ mice was infected with *A. fumigatus* for 3 days which was detected by Western blot and analyzed by imager systems described in Section 2 (*n* = 3/group). CD4^+^ T cells were sorted from the spleens of wild-type C57BL/6 mice and cultured. Tregs were detected by flow cytometry on day 3 and analyzed by FlowJo10. (d) CD4^+^ T lymphocytes differentiated fewer CD4^+^CD25^+^ T cells and CD4^+^CD25^+^Foxp3^+^ Tregs after treatment with C29. In each group, *n* = 5; three replicate experiments were performed three times. ns: not significant; ^∗^*p* < 0.05, ^∗∗^*p* < 0.01, ^∗∗∗^*p* < 0.001, and ^∗∗∗∗^*p* < 0.0001 by the one-way ANOVA and the two-way ANOVA followed by the Tukey post hoc test. Error bars represent SEM.

To investigate the molecular mechanisms of Treg cell differentiation and proliferation caused by TLR2 in *A. fumigatus* infection, as shown in Figure 4(a), the ratios of the CD25^+^ Tregs (*p* < 0.01) and CD25^+^Foxp3^+^ Tregs (*p* < 0.0001) in CD4^+^ T cells of the spleen of TLR2^−/−^ mice were significantly lower than those in the WT mice. After *A. fumigatus* infection, the CD25^+^ Tregs (*p* < 0.05) and CD25^+^Foxp3^+^ Tregs (*p* < 0.01) in CD4^+^ T cells of the spleen of TLR2^−/−^ mice were significantly also lower. The results are in line with previous findings [11]. We also detected the expression of Foxp3 in the lungs of mice after infection with *A. fumigatus*. RT-PCR results showed that the expression of Foxp3 in the lungs of the control group TLR2^−/−^ mice was reduced compared to that of wild-type mice (P <0.001, Figure 4(b)). The expression of Foxp3 was decreased in the lung of no-infection TLR2^−/−^ mice compared with WT mice in control (*p* < 0.05). Besides, although the expression of Foxp3 was upregulated after infection with *A. fumigatus* in the lung of TLR2^−/−^ mice and WT mice, those of TLR2^−/−^ mice were also reduced compared to WT mice (*p* < 0.05, Figure 4(c)).

### 3.7. The Inhibitor of TLR2 Reduces *Aspergillus fumigatus*-Induced CD4^+^ CD25^+^ Treg Cell Differentiation in CD4^+^ T Lymphocytes

To confirm whether TLR2 in *A. fumigatus* infection can affect the differentiation of CD4^+^ T lymphocytes into Treg cells, we obtained primary CD4^+^ T lymphocytes from mouse spleens for subsequent in vitro cell culture experiments. Intervention was done by adding C29 (TLR2 inhibitor) to the medium. It was observed that flow cytometry analysis was performed after 72 hours of culture, and we found that the ratio of CD4^+^ T lymphocytes differentiated into CD25^+^ Tregs and CD25^+^Foxp3^+^ Tregs decreased after C29 treatment (*p* < 0.0001, Figure 4(d)).

### 3.8. TRL2 Signaling Involves GSDMD-Dependent Pyroptosis in *Aspergillus fumigatus*

**Figure 5:**
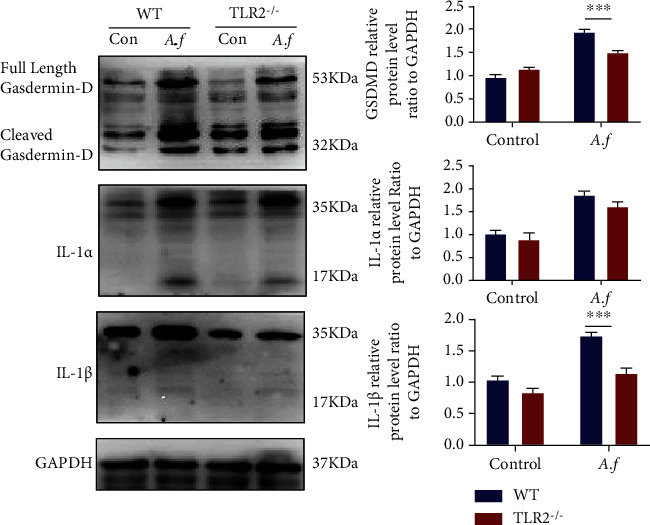
TRL2 signaling involves GSDMD-dependent pyroptosis in *Aspergillus fumigatus.* The expression of GSDMD, IL-1*α*, and IL-1*β* protein in lungs was detected by Western blot and analyzed by imager systems described in Section 2 (*n* = 3/group). ns: not significant; ^∗^*p* < 0.05, ^∗∗^*p* < 0.01, ^∗∗∗^*p* < 0.001, and ^∗∗∗∗^*p* < 0.0001 by two-way ANOVA followed by the Tukey post hoc test comparing the WT, WT + *A*.*f*., and TLR2^−/−^, TLR2^−/−^ + *A*.*f*. groups. Error bars represent SEM.

Gasdermin-D-dependent pyrolysis signal molecules play an important role in lung damage caused by infection [28]. We infected the lungs of TLR2^−/−^ mice and WT mice with 1 × 10^7^ conidia and evaluated GSDMD, IL-1*α*, and IL-*β* proteins by Western blot. As shown in Figure 5, GSDMD, IL-1*α*, and IL-*β* proteins in lung tissue were induced after *A. fumigatus* stimulation, whether WT mice or TLR2 knockout mice. Besides, the expression of both IL1-*β* and GSDMD in TLR2^−/−^ mice decreased compared with that in WT mice (*p* < 0.001).

## 4. Discussion

*A. fumigatus* can cause a wide range of diseases, from hypersensitivity to invasive infection. *A. fumigatus* usually occurs in critical patients, which is accompanied by severely immunocompromised and prolonged neutropenia mainly. Although IA has been considered a rare condition among critically ill patients, recent data indicate high incidence and should be reconsidered as an emerging and devastating infectious disease in ICU patients. The lung was the most frequent site of infection (94%), and *Aspergillus fumigatus* is the most commonly isolated species (92%) [29]. IA due to *A. fumigatus* is associated with greater severity, high mortality, and more frequent organ support.

Currently, therapy for diseases (such as cancer and autoimmune disease), based on immune escape mechanisms, has become increasingly attractive in the biomedical field. And there are dynamic and complex interactions between the host and *A. fumigatus* [30]. Immune recognition, escaping immune recognition, and counteracting host responses constitute the series of mechanisms after *A. fumigatus* invades the host. Although inflammation is primarily a defense reaction with detrimental consequences to the pathogen, its downstream effects, such as changes in the metabolism or influx of immune cells, might actually favor the growth and tissue spread of the pathogen. Microbe-directed skewing of the immune response by specific signals might further diminish the antimicrobial effect and enhance the pathogen's benefit [31]. In the pathogen infection, Tregs although prevent infection-associated inflammation and tissue damage also dampen the protective immune response to pathogens and enhance their persistence [32]. Tregs that specifically target *A. fumigatus* have been described in humans [33] and mice [9]. Our study identified that IL-1*β*, IL-6, and IL-2R were significantly elevated in clinical biological samples of *A. fumigatus* patients (Figure 1(a)). In mice after *A. fumigatus* infection of the lung, data showed that lung damaged and increased cytokines in mice with *A. fumigatus* infection, too (Figures 1(b)–1(d)). However, the ratio of CD4^+^CD25^+^Foxp3^+^ Tregs was heightened after *A. fumigatus* infection in patients (Figure 2(a)). And we found that the levels of CD4^+^CD25^+^Foxp3^+^ Tregs in the spleen and Foxp3 expression in the lung were increased after *A. fumigatus* infection (Figures 2(b) and 2(c)). Tregs have been proven to inhibit inflammation. So what role does the anti-inflammatory effect of Tregs play on host *A. f.* infection in a normal immune state? What is the significance of the increase in Tregs, which is synchronized with the increase of lung injury and inflammatory factor expression, after lung injury induced by *A. fumigatus* infection? Is it a response to the anti-inflammatory effects or other possible values? To further understand the relationship between Tregs and fungal persistence, we use CD25-neutralizing antibody to inhibit Tregs (Figure 2(d)) and found that the number of fungal burden in the lung was decreased in mice treated with CD25-neutralizing antibody (Figure 2(e)). The study showed that CD4^+^CD25^+^Foxp3^+^ Tregs are not generated in B7-2^−^ or CD28^−^ deficient mice, these mice are capable of efficiently restricting the fungal growth [34]. In *A. fumigatus* infection, the fungal burden was higher and the inflammatory tissue pathology was milder in WT than in CD4^+^CD25^+^Foxp3^+^ Treg-reduced mice [9]. Immune dysfunction contributes to worse outcomes of pathogenic microorganism infection. Partial depletion of Tregs elevated IL-17A, IL-1*β*, and IL-6 production and decreased IL-10 levels, leading to lower bacterial load and attenuation of lung injury in secondary *P. aeruginosa* infection after sepsis [35]. The deleterious role of Tregs on the innate immune response was underscored in the improved resistance to *C. albicans* infection [16]. Our results show that the lung could be partially rescued after depletion of Tregs (Figure 2(f)). These results could imply that the effects of Treg cells are deleterious when a pathogen, such as *A. fumigatus*, is persistent.

Along with *A. fumigatus* infection developing, TLRs trigger antimicrobial host immune responses. TLR2, as an important pathogen pattern recognition receptor, plays a vital role in infection [10, 24]. It is an important receptor able to recognize the hypha and spores of *A. fumigatus* [17]. Our study found that *A. fumigatus* infection can indeed stimulate TLR2 expression to increase (Figure 3(a)). We observed that the susceptibility of TLR2-deficient mice to *A. fumigatus* was not different from that of controls, a finding suggesting that the mice are fully competent at the level of innate antifungal resistance, as documented by reduced fungal growth in mice with primary disseminated candidiasis [36]. Interestingly, 3 days after *A. fumigatus* infection, we observed that the fungal burden and injury in TLR2^−/−^ mouse lungs were decreased compared to those in controls (Figures 3(b) and 3(c)). It implies that *A. fumigatus* could evade host defense through TLR2-mediated signals probably. And the expression of TNF-*α* and IL-6 was marginally impaired in TLR2^−/−^ mice (Figure 3(d)). It suggested that TLR4 [37], C-type lectin receptors (CLRs) [38–40], and galectin family proteins involved in *A. fumigatus*-induced proinflammatory cytokine release, too. Different TLRs may modulate the adaptive immune response through either stimulation or inhibition of Treg cell functions. Accumulated evidence has demonstrated that CD4^+^Foxp3^+^ Tregs can sense pathogens and modify their behavior through TLRs [41]. Previous studies have shown that CD4^+^Foxp3^+^ Tregs can express an array of several TLR mRNA, including TLR1, 2, 4, 5, 6, 7, and 8 but stimulation of only a few TLRs (such as TLR2, TLR5, and TLR8) affects the proliferation and/or suppressive function of CD4^+^Foxp3^+^ Tregs [42, 43]. IL-10 induces the development of CD4^+^ Tregs in a costimulation- and TLR-dependent fashion in fungus infection [44–46]. The study shows that *C. albicans* induces immunosuppression through TLR2-derived signals that mediate increased IL-10 production and Treg cells' survival [16]. And another evidence showed that dengue infection induced the proliferation of functional CD4^+^Foxp3^+^ Tregs via the TLR2/MyD88 pathway [11]. Our study found that TLR2-deficient mice have a significant decrease in Tregs of the spleen (Figure 4(a)) and the expression of Foxp3 of the lung (Figures 4(b) and 4(c)). On the other hand, starting from naive cells, CD4^+^ T cells can differentiate into various effector cell subsets with specialized functions. Tregs show strong plasticity allowing the functional adaptation to various physiological and pathological environments during immune responses [47]. TLR signaling is involved in T cell population regulation [16, 48]. After inhibiting the TLR2 pathway, the differentiation of Tregs from CD4^+^ T cells promoted by *A. fumigatus* stimulation decreased (Figure 4(d)). It suggests that TLR2-mediated signals are crucial for the generation of Treg cells. The present data prompt that *A. fumigatus* infection induced the proliferation of CD4^+^CD25^+^Foxp3^+^ Tregs via the activation of the TLR2 pathway.

*A. fumigatus* produces an abundance of spores, which are able to activate multiple inflammasomes [49]. It can lead to the host's inflammasome activation, causing the activation of the pyroptosis pathway [50]. Evidence suggests that fungal DNA, spores, and cell wall-associated polysaccharides are recognized by inflammasome sensors [51, 52], which often leads to activation of a cytosolic macromolecular signaling platform that mediates the release of the proinflammatory cytokines IL-1 and IL-18 and cleavage of the pore-forming protein Gasdermin-D (GSDMD). Pyroptosis is a highly proinflammatory event because the proform of IL-1*β* is processed by inflammasome-dependent caspase-1 activation and released during cell death [53]. In previous studies, immunocompetent WT mice and mice lacking the inflammasome components like NLRP3 or absent in melanoma 2(AIM2) do not succumb to infection with *A. fumigatus* [51]. Our research selected *A. fumigatus* to infect WT and TLR2^−/−^ mice, with immunocompetence. Then, we found that the expression of GSDMD, IL-1*α*, and IL-1*β* increased in WT mice after lung infection of *A. fumigatus* (Figure 5). The NLRP3 inflammasome in monocytes is stimulated by *A. fumigatus*, and hyphae upregulate pro-IL-1*β* expression and induce IL-1*β* secretion in human monocytes [49]. Studies showed that the expression of NLRP3 was increased in lung tissue from patients with allergic bronchopulmonary aspergillosis (ABPA) [54]. And inflammasome-mediated IL-1*β* secretion requires some steps, including the engagement of TLR signaling via proinflammatory stimuli, induction the proform of cytokines, and activation of the inflammasome promoting mature cytokine processing [41]. IL-1*β* mRNA was partially reduced in TLR2^−/−^ compared with WT macrophages during *C. difficile* infection [55]. *H. pylori* activates the inflammasome in a TLR2- and NLRP3-dependent manner, and *H. pylori* benefits from inflammasome activation, which ensures persistent infection [56]. In TLR2^−/−^ mice, the pyrolysis-related proteins (GSDMD, IL-1*α*, and IL-1*β*) upregulated, which showed the immunocompetence in the infection of *A. fumigatus*. But they were decreased after the infection of *A. fumigatus*, compared with WT mice (Figure 5). Although the intracellular receptor that engages inflammasome activation and the physiological function of the inflammasomes in response to *A. fumigatus* infection remain to be elucidated, our results provided preliminary evidence to suggest that TRL2 plays a role in GSDMD-dependent pyrolysis of the lung after *A. fumigatus* infection partially.

## 5. Conclusion

Susceptibility to *A. fumigatus* is associated with the quantity of CD4^+^CD25^+^Foxp3^+^ Tregs in TLR2 knockout animals. The infection leads to the proliferation and differentiation of CD4^+^CD25^+^Foxp3^+^ Tregs via the activation of the TLR2 pathway. It is a potential mechanism to evade host defense in *A. fumigatus* infection of the lung. And this effect can regulate GSDMD-dependent pyroptosis and may involve TRL2 signals partially.

## Data Availability

The data used to support the findings of this study are included within the article.

## References

[1] Sales-Campos H., Tonani L., Cardoso C. R. B., Kress M. R. V. Z. (2013). The immune interplay between the host and the pathogen in Aspergillus fumigatus lung infection. *BioMed Research International*.

[2] Latge J. P., Chamilos G. (2019). Aspergillus fumigatus and Aspergillosis in 2019. *Clinical Microbiology Reviews*.

[3] Kosmidis C., Denning D. W. (2015). The clinical spectrum of pulmonary aspergillosis. *Thorax*.

[4] Latge J. P. (2001). The pathobiology of _Aspergillus fumigatus_. *Trends in Microbiology*.

[5] Brown G. D., Denning D. W., Gow N. A. R., Levitz S. M., Netea M. G., White T. C. (2012). Hidden killers: human fungal infections. *Science Translational Medicine*.

[6] Kontoyiannis D. P., Marr K. A., Park B. J. (2010). Prospective surveillance for invasive fungal infections in hematopoietic stem cell transplant recipients, 2001-2006: overview of the Transplant-Associated Infection Surveillance Network (TRANSNET) database. *Clinical Infectious Diseases*.

[7] Wang F., Zhang C., Jiang Y. (2017). Innate and adaptive immune response to chronic pulmonary infection of hyphae of Aspergillus fumigatus in a new murine model. *Journal of Medical Microbiology*.

[8] Bacher P., Kniemeyer O., Teutschbein J. (2014). Identification of immunogenic antigens from Aspergillus fumigatus by direct multiparameter characterization of specific conventional and regulatory CD4+ T cells. *Journal of Immunology*.

[9] Montagnoli C., Fallarino F., Gaziano R. (2006). Immunity and tolerance to Aspergillus involve functionally distinct regulatory T cells and tryptophan catabolism. *Journal of Immunology*.

[10] Stephen-Victor E., Karnam A., Fontaine T. (2017). Aspergillus fumigatus cell wall alpha-(1,3)-glucan stimulates regulatory T-cell polarization by inducing PD-L1 expression on human dendritic cells. *The Journal of Infectious Diseases*.

[11] George J. A., Park S. O., Choi J. Y., Uyangaa E., Eo S. K. (2020). Double-faced implication of CD4(+) Foxp3(+) regulatory T cells expanded by acute dengue infection via TLR2/MyD88 pathway. *European Journal of Immunology*.

[12] Jie Z., Wu X., Yu F. S. (2009). Activation of Toll-like receptors 2 and 4 in Aspergillus fumigatus keratitis. *Innate Immunity*.

[13] Medzhitov R. (2001). Toll-like receptors and innate immunity. *Nature Reviews. Immunology*.

[14] Kulkarni R., Behboudi S., Sharif S. (2011). Insights into the role of Toll-like receptors in modulation of T cell responses. *Cell and Tissue Research*.

[15] Dai J., Liu B., Li Z. (2009). Regulatory T cells and Toll-like receptors: what is the missing link?. *International Immunopharmacology*.

[16] Netea M. G., Sutmuller R., Hermann C. (2004). Toll-like receptor 2 suppresses immunity against Candida albicans through induction of IL-10 and regulatory T cells. *Journal of Immunology*.

[17] Netea M. G., Warris A., Van der Meer J. W. (2003). Aspergillus fumigatus evades immune recognition during germination through loss of Toll-like receptor-4-mediated signal transduction. *The Journal of Infectious Diseases*.

[18] Mirkov I., Demenesku J., Popov Aleksandrov A. (2015). Strain differences in the immune mechanisms of resistance of immunocompetent rats to pulmonary aspergillosis. *Immunobiology*.

[19] Dai J., Liang Y., Li H. (2018). Vitamin D enhances resistance to aspergillus fumigatus in mice via inhibition of excessive autophagy. *American Journal of Translational Research*.

[20] Rivera A., Hohl T. M., Collins N. (2011). Dectin-1 diversifies Aspergillus fumigatus-specific T cell responses by inhibiting T helper type 1 CD4 T cell differentiation. *The Journal of Experimental Medicine*.

[21] Chai Y. S., Chen Y. Q., Lin S. H. (2020). Curcumin regulates the differentiation of naive CD4+T cells and activates IL-10 immune modulation against acute lung injury in mice. *Biomedicine & Pharmacotherapy*.

[22] Mikawa K., Nishina K., Takao Y., Obara H. (2003). ONO-1714, a nitric oxide synthase inhibitor, attenuates endotoxin-induced acute lung injury in rabbits. *Anesthesia and Analgesia*.

[23] Cimolai N., Taylor G. P., Mah D., Morrison B. J. (1992). Definition and application of a histopathological scoring scheme for an animal model of acute mycoplasma pneumoniae pulmonary infection. *Microbiology and Immunology*.

[24] Raijmakers R. P. H., Sprenkeler E. G. G., Aleva F. E. (2017). Toll-like receptor 2 induced cytotoxic T-lymphocyte-associated protein 4 regulates Aspergillus-induced regulatory T-cells with pro-inflammatory characteristics. *Scientific Reports*.

[25] Flaherty S., Reynolds J. M. (2015). Mouse Naïve CD4^+^ T cell isolation and *In vitro* differentiation into T cell subsets. *Journal of Visualized Experiments*.

[26] Grabowski M., Murgueitio M. S., Bermudez M., Wolber G., Weindl G. (2020). The novel small-molecule antagonist MMG-11 preferentially inhibits TLR2/1 signaling. *Biochemical Pharmacology*.

[27] Liu C., Wang M., Sun W. (2017). PU.1 serves a critical role in the innate defense against Aspergillus fumigatus via dendritic cell-associated C-type lectin receptor-1 and toll-like receptors-2 and 4 in THP-1-derived macrophages. *Molecular Medicine Reports*.

[28] Liu X., Lieberman J. (2017). A mechanistic understanding of pyroptosis: the fiery death triggered by invasive infection. *Advances in Immunology*.

[29] Taccone F. S., Van den Abeele A. M., Bulpa P. (2015). Epidemiology of invasive aspergillosis in critically ill patients: clinical presentation, underlying conditions, and outcomes. *Critical Care*.

[30] Stewart J. I. P., Fava V. M., Kerkaert J. D. (2020). Reducing Aspergillus fumigatus virulence through targeted dysregulation of the conidiation pathway. *mBio*.

[31] Flieger A., Frischknecht F., Haecker G., Hornef M. W., Pradel G. (2018). Pathways of host cell exit by intracellular pathogens. *Microbial Cell*.

[32] Stephen-Victor E., Bosschem I., Haesebrouck F., Bayry J. (2017). The Yin and Yang of regulatory T cells in infectious diseases and avenues to target them. *Cellular Microbiology*.

[33] Bedke T., Iannitti R. G., De Luca A. (2014). Distinct and complementary roles for Aspergillus fumigatus-specific Tr1 and Foxp3+ regulatory T cells in humans and mice. *Immunology and Cell Biology*.

[34] Montagnoli C., Bacci A., Bozza S. (2002). B7/CD28-dependent CD4+CD25+ regulatory T cells are essential components of the memory-protective immunity to Candida albicans. *Journal of Immunology*.

[35] Hu Z. Q., Yao Y. M., Chen W. (2018). Partial depletion of regulatory T cells enhances host inflammatory response against acute Pseudomonas aeruginosa infection after Sepsis. *Inflammation*.

[36] Bellocchio S., Montagnoli C., Bozza S. (2004). The contribution of the Toll-like/IL-1 receptor superfamily to innate and adaptive immunity to fungal pathogens in vivo. *Journal of Immunology*.

[37] Taghavi M., Khosravi A., Mortaz E., Nikaein D., Athari S. S. (2017). Role of pathogen-associated molecular patterns (PAMPS) in immune responses to fungal infections. *European Journal of Pharmacology*.

[38] Werner J. L., Metz A. E., Horn D. (2009). Requisite role for the dectin-1 beta-glucan receptor in pulmonary defense against Aspergillus fumigatus. *Journal of Immunology*.

[39] Gessner M. A., Werner J. L., Lilly L. M. (2012). Dectin-1-dependent interleukin-22 contributes to early innate lung defense against Aspergillus fumigatus. *Infection and Immunity*.

[40] Taylor P. R., Roy S., Leal S. M. (2014). Activation of neutrophils by autocrine IL-17A-IL-17RC interactions during fungal infection is regulated by IL-6, IL-23, RORgammat and dectin-2. *Nature Immunology*.

[41] Zanin-Zhorov A., Cohen I. R. (2013). Signaling via TLR2 and TLR4 directly down-regulates T cell effector functions: the regulatory face of danger signals. *Frontiers in Immunology*.

[42] Liu H., Komai-Koma M., Xu D., Liew F. Y. (2006). Toll-like receptor 2 signaling modulates the functions of CD4+ CD25+ regulatory T cells. *Proceedings of the National Academy of Sciences of the United States of America*.

[43] Sutmuller R. P., den Brok M. H., Kramer M. (2006). Toll-like receptor 2 controls expansion and function of regulatory T cells. *The Journal of Clinical Investigation*.

[44] Mills K. H. (2004). Regulatory T cells: friend or foe in immunity to infection?. *Nature Reviews Immunology*.

[45] Belkaid Y., Rouse B. T. (2005). Natural regulatory T cells in infectious disease. *Nature Immunology*.

[46] O'Garra A., Vieira P. (2004). Regulatory T cells and mechanisms of immune system control. *Nature Medicine*.

[47] Wan Z., Zhou Z., Liu Y. (2020). Regulatory T cells and T helper 17 cells in viral infection. *Scandinavian Journal of Immunology*.

[48] Rau C. S., Lin M. W., Wu S. C. (2015). Regulatory and effector helper T-cell profile after nerve xenografting in the Toll-like receptor-deficient mice. *International Journal of Medical Sciences*.

[49] Said-Sadier N., Padilla E., Langsley G., Ojcius D. M. (2010). Aspergillus fumigatus stimulates the NLRP3 inflammasome through a pathway requiring ROS production and the Syk tyrosine kinase. *PLoS One*.

[50] van de Veerdonk F. L., Joosten L. A., Netea M. G. (2015). The interplay between inflammasome activation and antifungal host defense. *Immunological Reviews*.

[51] Karki R., Man S. M., Malireddi R. K. S. (2015). Concerted activation of the AIM2 and NLRP3 inflammasomes orchestrates host protection against *Aspergillus* infection. *Cell Host & Microbe*.

[52] Huang Y., Hua M., Cui X. (2018). Fungal beta-glucan activates the NLRP3 inflammasome in human bronchial epithelial cells through ROS production. *Inflammation*.

[53] Miao E. A., Leaf I. A., Treuting P. M. (2010). Caspase-1-induced pyroptosis is an innate immune effector mechanism against intracellular bacteria. *Nature Immunology*.

[54] Jeong J. S., Lee K. B., Kim S. R. (2018). Airway epithelial phosphoinositide 3-kinase-delta contributes to the modulation of fungi-induced innate immune response. *Thorax*.

[55] Liu Y. H., Chang Y. C., Chen L. K. (2018). The ATP-P2X7 signaling axis is an essential sentinel for intracellular Clostridium difficile pathogen-induced Inflammasome activation. *Frontiers in Cellular and Infection Microbiology*.

[56] Koch K. N., Hartung M. L., Urban S. (2015). Helicobacter urease-induced activation of the TLR2/NLRP3/IL-18 axis protects against asthma. *The Journal of Clinical Investigation*.

